# Untargeted Metabolomics to Go beyond the Canonical Effect of Acetylsalicylic Acid

**DOI:** 10.3390/jcm9010051

**Published:** 2019-12-24

**Authors:** Alessandro Di Minno, Benedetta Porro, Linda Turnu, Chiara Maria Manega, Sonia Eligini, Simone Barbieri, Mattia Chiesa, Paolo Poggio, Isabella Squellerio, Andrea Anesi, Susanna Fiorelli, Donatella Caruso, Fabrizio Veglia, Viviana Cavalca, Elena Tremoli

**Affiliations:** 1 Dipartimento di Farmacia, Università degli Studi di Napoli Federico II, 80131 Naples, Italy; alessandro.diminno@unina.it; 2 Centro Cardiologico Monzino IRCCS, Unit of Metabolomics and Cellular Biochemistry of Atherothrombosis, 20138 Milan, Italy; bporro@ccfm.it (B.P.); linda.1987@libero.it (L.T.); chiara.manega@unimi.it (C.M.M.); seligini@ccfm.it (S.E.); isquellerio@ccfm.it (I.S.); sfiorelli@ccfm.it (S.F.); 3 Centro Cardiologico Monzino IRCCS, Unit of Biostatistics, 20138 Milan, Italy; simone.barbieri@ccfm.it (S.B.); fveglia@ccfm.it (F.V.); 4 Centro Cardiologico Monzino IRCCS, Unit of Immunology and Functional Genomics, 20138 Milan, Italy; mchiesa@ccfm.it; 5 Centro Cardiologico Monzino IRCCS, Unit for the Study of Aortic, Valvular and Coronary Pathologies, 20138 Milan, Italy; ppoggio@ccfm.it; 6 Department of Food Quality and Nutrition, Research and Innovation Centre, Fondazione Edmund Mach, 38010 San Michele all’Adige, Italy; andrea.anesi@fmach.it; 7 Dipartimento di Scienze Farmacologiche e Biomolecolari, Università degli Studi di Milano, 20122 Milan, Italy; donatella.caruso@unimi.it; 8 Centro Cardiologico Monzino IRCCS, 20138 Milan, Italy; etremoli@ccfm.it

**Keywords:** aspirin, metabolomics, untargeted analysis, glutamine

## Abstract

Given to its ability to irreversibly acetylate the platelet cyclooxygenase-1 enzyme, acetylsalicylic acid (ASA) is successfully employed for the prevention of cardiovascular disease. Recently, an antitumoral effect of ASA in colorectal cancer has been increasingly documented. However, the molecular and metabolic mechanisms by which ASA exerts such effect is largely unknown. Using a new, untargeted liquid chromatography–mass spectrometry approach, we have analyzed urine samples from seven healthy participants that each ingested 100 mg of ASA once daily for 1 week. Of the 2007 features detected, 25 metabolites differing after ASA ingestion (nominal *p* < 0.05 and variable importance in projection (VIP) score > 1) were identified, and pathway analysis revealed low levels of glutamine and of metabolites involved in histidine and purine metabolisms. Likewise, consistent with an altered fatty acid *β*-oxidation process, a decrease in several short- and medium-chain acyl-carnitines was observed. An abnormal *β*-oxidation and a lower than normal glutamine availability suggests reduced synthesis of acetyl-Co-A, as they are events linked to one another and experimentally related to ASA antiproliferative effects. While giving an example of how untargeted metabolomics allows us to explore new clinical applications of drugs, the present data provide a direction to be pursued to test the therapeutic effects of ASA—e.g., the antitumoral effect—beyond cardiovascular protection.

## 1. Introduction

Aspirin, the brand name of acetylsalicylic acid (ASA), is the most commonly used nonsteroidal anti-inflammatory drug in the world. It is administrated at low doses in the long-term prevention of cardiovascular disease (CVD) [1], and its use in counteracting the long-term risk of death due to cancer has been recently suggested [2,3]. The “canonical” effect of ASA is due to the irreversible acetylation of the platelet cyclooxygenase (COX)-1 enzyme, and in turn to the long-term suppression of the synthesis of thromboxane (TX) A_2_, a potent platelet agonist derived from the metabolism of arachidonic acid [4].

Non-COX-1 mediated effects of ASA that may contribute to explaining its antithrombotic and anti-inflammatory action, as well as other anti-atherosclerotic properties, are increasingly recognized [5,6]. In vitro cell models have shown that ASA and its metabolite salicylic acid induce the expression of some mitochondrial respiratory genes, as well as of genes involved in cholesterol homeostasis, showing new beneficial effects of ASA in atherosclerosis [6,7]. Modulation of the adenosine monophosphate-activated protein kinase (AMPK) signaling pathway in cellular models has also been shown [6,8].

In order to explore the non-canonical ASA effects, we took advantage of pharmaco-metabolomics, an emerging area of research that helps to define the mechanism of action of drugs and individual responses to drug treatment through the analysis of metabolites [9,10]. The study of low-molecular-weight metabolite levels allows to investigate drug effects in biological fluids (e.g., urine or blood) as the result of changes arising in response to treatment. This also lets us highlight the biochemical pathways involved in drug effects, and in turn amplifies the spectrum of drug application [11,12].

In this pilot study, an untargeted metabolomic approach by liquid chromatography–quadrupole time-of-flight mass spectrometry (LC-QTOF-MS) on urine of healthy participants, before and after 7 days of low-dose ASA ingestion, was applied to a broad-spectrum appraise the metabolic profiles affected by ASA and potentially involved in cardiovascular and cancer prevention.

## 2. Materials and Methods

### 2.1. Chemicals and Reagents

The purity of solvents, reagents, and chemical standards is reported in Appendix A.

### 2.2. Study Design

Seven healthy participants (HPs; aged 29.9 ± 2.6 years; 42.8% men) were enrolled in the present pilot study at the Centro Cardiologico Monzino, IRCCS (CCM). Participants ingested low-dose, enteric-coated 100 mg ASA once-daily (od) for 7 consecutive days. A 24 h urine sample collection was obtained before the first (T0) and after the last (T7) ASA intake. Serum samples were obtained at T0 and T7 after 2 h of incubation at 37 °C. All participants were instructed to avoid other drugs during the study week.

The present study, approved by the CCM Institutional Ethics Committee (n° CCM 525), was carried out according to the ethical guidelines of the 1975 Declaration of Helsinki. Written informed consent to participate was obtained from all participants.

### 2.3. COX-mediated Effect of Acetylsalicylic Acid: TXA_2_ Metabolite Measurement

The COX-1 mediated effect of ASA was assessed through the measurement of serum levels of TXB_2_, the stable metabolite of TXA_2_, by a previously developed and validated liquid chromatography–tandem mass spectrometry (LC-MS/MS) method [13]. In addition, as an index of systemic TXA_2_ biosynthesis, the urinary TXA_2_ catabolic metabolite 11-dehydro thromboxane B_2_ (11-dehydro TXB_2_), was also measured [14].

### 2.4. LC-QTOF-MS Metabolic Fingerprinting

#### 2.4.1. Sample Preparation

Twenty-four hour urine samples were obtained from all study participants; during the 24 h sample collection, urine was maintained at 4 °C. Once collection has been completed, urine was aliquoted and stored at −80 °C until analysis. Frozen urine was thawed at room temperature and then centrifuged at 1700× *g* for 10 min. The supernatant (100 µL) was diluted with a reference standard water solution containing 11-dehydro-thromboxane B2-d_4_ (11-DH-TXB_2_-d_4_), 8-iso-prostaglandin F_2α_-d_4_ (8-iso-PGF_2α_-d_4)_, 12-hydroxyeicosatetraenoic acid-d_8_ (12-HETE-d_8_), reserpine, 3-nitro-tyrosine-^13^C_9_ (3-nitro-tyr-^13^C_9_), 8-hydroxy-2-deoxyguanosine-^15^N_5_ (8-OHdG-^15^N_5_), and salicylic acid-d_4_ (SA-d_4_) (100 µL, final concentration 1 ng/mL each). Samples were centrifuged at 12,000× *g* for 20 min and aliquoted in two vials for the LC-QTOF-MS metabolic fingerprinting and the liquid chromatography–quadrupole time-of-flight–tandem mass spectrometry (LC-QTOF-MS/MS) analysis.

#### 2.4.2. Ultra-High-Performance LC-QTOF-MS Method Sample Analysis

LC-QTOF-MS analysis was performed by an ultra-high-performance liquid chromatography system (1290 Infinity series, Agilent Technologies, Santa Clara, CA, USA), coupled to a quadrupole time-of-flight mass spectrometry detector (Agilent 6550 iFunnel Q-TOF) outfitted with an electrospray ionization (ESI) source. Samples were analyzed in duplicate, both in positive and in negative detection mode. One µL was injected onto the Zorbax Eclipse Plus C18 reverse phase column (2.1 × 150 mm, 1.8 µm, Agilent Technologies), maintained at 40 °C. For the positive ion mode run, the used mobile phases were: water + acetonitrile (95:5 v/v) with 0.1% formic acid (A) and acetonitrile with 0.1% formic acid (B). The gradient was as follows: 2 min with 100% A, 2–12 min with 100–95% A, 12–18 min with 95–0% A, 18–22 min with 0% A, and 22–23 min with 0–100% A. For the negative ion mode run, mobile phase A was ammonium acetate 10 mM, and mobile phase B was acetonitrile with ammonium acetate 10 mM. The gradient was as follows: 2 min with 99% A, 2–12 min with 99–95% A, 12–18 min with 95–0% A, 18–22 min with 0% A, and 22–23 min with 0–99% A. Both analyses were conducted at a constant flow rate of 0.4 mL/min. The detector operated in full scan mode, acquiring mass spectra over the *m*/*z* range of 40–1100 Da, with a scan rate of 2 scans per second. The source parameters for both ion modes were drying gas temperature (280 °C), drying gas flow (13 l/min), nebulizer pressure (45 psig), sheath gas temperature (300 °C), sheath gas flow (12 l/min), capillary voltage (3000 V), nozzle voltage (500 V), and fragmentor (150 V). The reference masses were 121.0509 *m*/*z* and 922.0098 *m*/*z* (ESI+), and 112.9856 *m*/*z* and 1033.9881 *m*/*z* (ESI-). These were continuously infused to correct instrument variability. Data were acquired by Agilent’s MassHunter Workstation Data Acquisition software.

#### 2.4.3. Performance Evaluation

Performance evaluation was assessed by analyzing reference standards spiked in urine and quality control (QC) samples. QC samples were obtained by pooling together equal volumes of all urine samples (T0 + T7) spiked with the reference standard solution. Subsequently, QCs were injected at the beginning of the analytical sequence (*n* = 9), every six injections, and at the end of the analysis (*n* = 3).

#### 2.4.4. Data Processing

Raw acquired data were analyzed by MassHunter Profinder software (Agilent Technologies) using the “Batch Recursive Feature Extraction” algorithm that provides a list of features that represent possible metabolites, combining different information from co-eluting ions, such as charge state, isotopic distribution, presence of adducts, and dimers. The most relevant parameters selected for feature extraction were 500 counts for peak filter (to clean background noise); charge state limited to 2; allowed ion species: +H, +Na, and +K in positive ion mode, and −H, +Cl, and +CH_3_COO in negative ion mode; and neutral loss of water for both ion modes. Through the same software was performed the peak alignment, using a retention time (RT) window of 0% + 0.15 min and a mass window of 15 ppm + 2 mDa. To improve data quality, a manual feature evaluation procedure was performed: features present in the blank (water), with an RT lower than 0.7 min, or subjected to the carryover or reference standard solution’s peaks were excluded. The abundance values of duplicates were averaged, and missing values were manually assigned as half the lowest value detected in a single group if it is random [15], or as zero if the feature was absent in the whole group (T0 or T7). Urine creatinine normalization was also performed to control the variation in urine output. Furthermore, a filtering quality assurance procedure was applied, in order to deem as reliable those features present in at least 90% of the QCs with a coefficient of variation (CV) < 30% [16]. The robust lists finally obtained, for positive and negative modes, were defined as compound lists.

#### 2.4.5. Compound Identification

The metabolite identification was based on their measured accurate *m*/*z* values (10 ppm mass error window) and on the comparison of their acquired LC-QTOF-MS/MS spectra with those available on different databases, such as Metlin (http://metlin.scripps.edu), Kyoto Encyclopedia of Genes and Genomes (http://www.kegg.jp/kegg), Human Metabolome Database (http://www.hmdb.ca), and Personal Compound Database and Library (Agilent Technologies). The fragment elucidation, performed by MassHunter Molecular Structure Correlator (Agilent Technologies), the agreement between RT and compounds’ polarity, and the biological significance also contributed to define putative matches. Annotation or identification was determined following official classification defined by the Metabolomics Standard Initiative [17].

#### 2.4.6. Ultra-High-Performance LC-QTOF-MS/MS Method Sample Analysis

The LC-QTOF-MS/MS experiments were performed only for nominally significant compounds (T7 vs. T0), using the same chromatographic separation and ionization conditions previously described. Compounds were targeted using their *m*/*z* value (isolation width 4 Da) and RT (ΔRT 0.9 min), and data were collected applying two fixed collision energies, 10 and 40 eV. Moreover, samples with the highest intensity of each compound were analyzed to optimize the MS/MS spectra quality. Two µL of sample were injected, and 32 runs (11 in positive ion mode and 21 in negative ion mode) were performed in order to avoid fragmentation of significant compounds co-eluting in the same analysis. Subsequently, spectra were processed through MassHunter Qualitative software (Agilent Technologies).

### 2.5. Statistics

The compound lists obtained from positive and negative modes were treated independently, and values were log transformed before analysis. Using the lists obtained after the quality assurance procedure (1048 in positive ion mode and 959 in negative ion mode), we performed unsupervised principal component analysis (PCA) by Mass Profiler Professional software (Agilent Technologies), and orthogonal, partial-least-squares discriminant analysis (OPLS-DA) by the SIMCA statistical package. In OPLS-DA, in negative ionization mode we used one predictive and two orthogonal components, and in positive ionization mode we used one predictive and three orthogonal components. The criterion for inclusion of a new component in the model was based on the resultant increase in overall R2X (explained variation parameter). For cross validation, the leave-one-out method was employed. Metabolite values before and after treatment were compared by paired Student’s *t*-test. Due to the nature of this study, a small-sample pilot investigation intended as a model for future studies, a formal sample size calculation was not applied. Accordingly, feature selection has not been based on stringent criteria (such as *p*-values corrected for multiple testing) as required in large metabolomics studies. Instead, in order to highlight the best candidate metabolites, we adopted a criterion based on a “variable importance in projection” (VIP) score above one, and on a “nominal” *p*-value (i.e., the *t*-test *p*-value not corrected for multiple testing) below 0.05. Thus, the *p*-values referred to in the present study should be interpreted as an illustration of a procedure for selecting metabolites to be applied in future studies, and not as a criterion to evaluate the generalizability of our results. The heatmap and pathway analysis were performed using MetaboAnalyst, which is a web-based tool for the visualization of metabonomics. Pathway analysis was tested by the Fishers’ exact test. The identified metabolites were visualized through Cytoscape v3.7.1 [18], using the app Metscape v3.1.3. In particular, MetScape was used to provide the bioinformatic framework for the visualization and interpretation of our metabolomic data [19].

The compound lists obtained from positive and negative modes were treated independently for statistical analysis. Compound levels at T0 and T7 were log-transformed before analysis and were compared by paired Student’s *t*-test. Correlations between identified compounds were determined using the Pearson test. All calculations were computed with the aid of the SAS software package (Version 9.4 SAS Institute Inc., Cary, NC, USA).

## 3. Results

### 3.1. TXA_2_ Metabolite Measurement

At T7, serum TXB_2_ concentrations were lower (more than 98% decrease) than at T0 in each participant, (T0: 205.69 ± 77.27 ng/mL; T7: 1.48 ± 0.41 ng/mL; *p* < 0.001). In agreement with this data, a significant decrease in urinary 11-dehydro TXB_2_ levels was observed after ASA treatment (381.7 ± 131.4 and 95.5 ± 39.0 pg/mg creatinine, respectively; *p* = 0.002).

### 3.2. LC-QTOF-MS Metabolic Fingerprint

#### 3.2.1. LC-QTOF-MS Sample Analysis

**Figure 1:**
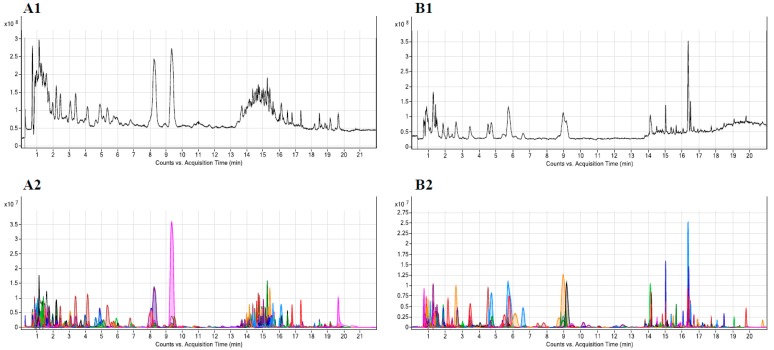
Representative chromatograms obtained from a urine sample: total ion chromatogram (TIC) in positive (**A1**) and negative (**B1**) detection modes; extracted compound chromatogram (ECC) in positive (**A2**) and negative (**B2**) detection modes.

To evaluate the metabolic modifications induced by ASA, we developed a LC-QTOF-MS untargeted method for urine sample analysis. Representative chromatograms, obtained in positive and negative ionization modes, are shown in Figure 1.

#### 3.2.2. Performance Evaluation

The reproducibility of sample preparation and analysis processes were valuated through two different approaches, based on QC samples and reference standards solution [20,21]. The total ion chromatogram (TIC) of QCs versus the order of injection showed good reproducibility in the whole analytical run, both for positive and negative ionization modes (CV: 1.64% and 1.62%, respectively). The TICs of sample replicates showed a very little intra-run variability (Figure S1, panels A1 and A2). Furthermore, the reproducibility of QC samples was confirmed using PCA score plots in both ionization modes (Figure S1, panels B1 and B2). In addition, the intensity of each reference standard, added to the sample before its preparation, was evaluated: CV values were lower than 10% for all the added standards, except for reserpine (Figure S1C).

#### 3.2.3. Data Processing

**Figure 2:**
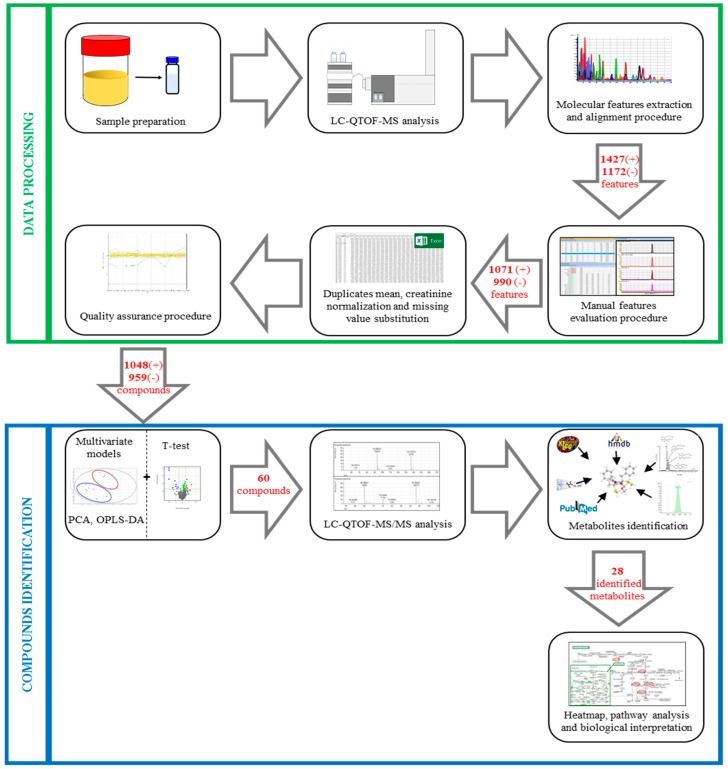
Representation of the untargeted workflow applied to analyze urine samples. In the green box are the steps involved in data processing; in the blue box are the steps regarding compound identification. In the arrows are reported the number of features or compounds obtained by the different steps, in positive (+) or negative (−) ionization modes. LC-QTOF-MS: Liquid Chromatography–Quadrupole Time-of-Flight–mass spectrometry; PCA: Principal Component Analysis; OPLS-DA: Orthogonal Partial-Least-Squares Discriminant Analysis; QTOF-MS/MS: Quadrupole Time of Flight-tandem mass spectrometry.

The workflow of data processing is depicted in Figure 2.

After data acquisition, a total of 2599 features (1427 in positive and 1172 in negative ionization modes) were extracted through the Batch Recursive Feature Extraction algorithm. After the visual validation of peak morphology and integration of compounds, the data set was reduced to 2061 (1071 and 990, respectively). Applying the quality assurance procedure, 2007 features were considered to be reliable compounds: 1048 for positive and 959 for negative modes. The compound lists (positive and negative) were used to build the multivariate models and to discriminate compounds potentially modified by ASA treatment.

The OPLS-DA model was built by positive and negative lists subtracted from ASA-related compounds and present only at T7. The plot of the first two OPLS-DA components, i.e., the two most relevant indices summarizing the data, is represented in Figure S2.

### 3.3. Compound Identification

#### Metabolic Changes Induced by ASA

The flow chart of compound identification is shown in Figure 2. In Supplementary Table S1 are reported all the metabolites that differ between T0 and T7 or those that are only present at T7 (*n* = 57). Sixty compounds differed between T0 and T7, while 11 metabolites are only present at T7; among them, we found ASA related metabolites, i.e., salicylic acid, 5-sulfosalicylic acid, and salicyluric acid. Based on the comparison of the acquired LC-QTOF-MS/MS spectra with those available on different databases, we identified 25 compounds. The identified metabolites with their relative fold change and VIP score are reported in Table 1.

### 3.4. Biological Interpretation

**Figure 3:**
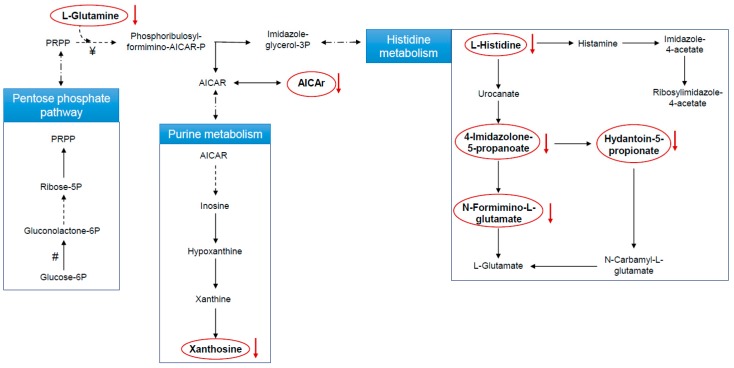
Schematic representation of histidine; alanine, aspartate, and glutamate; and purine metabolism (the main pathways affected by 7 days, low-dose acetylsalicylic acid (ASA) ingestion in healthy participants). Red circles highlight the metabolites putatively identified whose levels decrease at T7. #: Glucose-6P dehydrogenase; ¥: Glutamine-PRPP amidotransferase.

The identified metabolites were investigated, and the biochemical pathways in which they are involved were determined (Figure 3).

**Figure 4:**
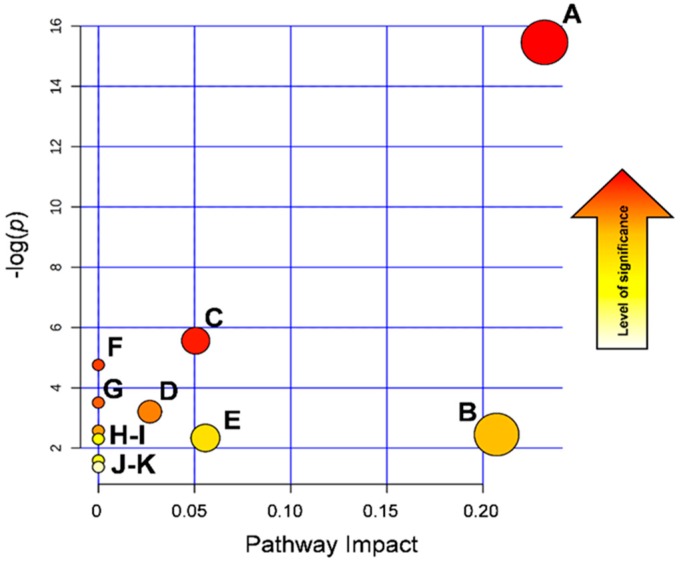
Pathway enrichment analysis showing the nodes in the graphic depicting the metabolic pathways affected by 7 day treatment with ASA 100 mg once daily. On the *y*-axis, the –log (*p*) value represents the quantitative perturbation of pathways. On the *x*-axis, the pathway impact value refers to the centrality of a metabolite in the metabolic network. The node color, varying from yellow to red, is based on its *p* value, and the node radius is determined on the basis of their pathway impact values. A: histidine metabolism; B: alanine, aspartate, and glutamate metabolism; C: purine metabolism, D: D-glutamine and D-glutamate metabolism; E: valine, leucine, and isoleucine biosynthesis, F: nitrogen metabolism; G: aminoacyl-tRNA biosynthesis; H: caffeine metabolism; I: β-alanine metabolism; J: pyrimidine metabolism; K: arginine and proline metabolism.

Eleven metabolic pathways were constructed, and among them, by applying the pathway enrichment analysis, histidine; alanine, aspartate, and glutamate; and purine metabolisms appeared to be the pathways maximally affected by the 7 days of exposure to low-dose ASA (Fishers’ exact test, *p* < 0.05, Figure 4).

The levels of several acylcarnitines, reflecting the β-oxidation process, were putatively affected by the low-dose ASA administration: in particular, the concentrations of butyryl-L-carnitine, tiglylcarnitine, isovalerylcarnitine, and heptanoylcarnitine were decreased, while those of methylglutarylcarnitine were increased.

**Figure 5:**
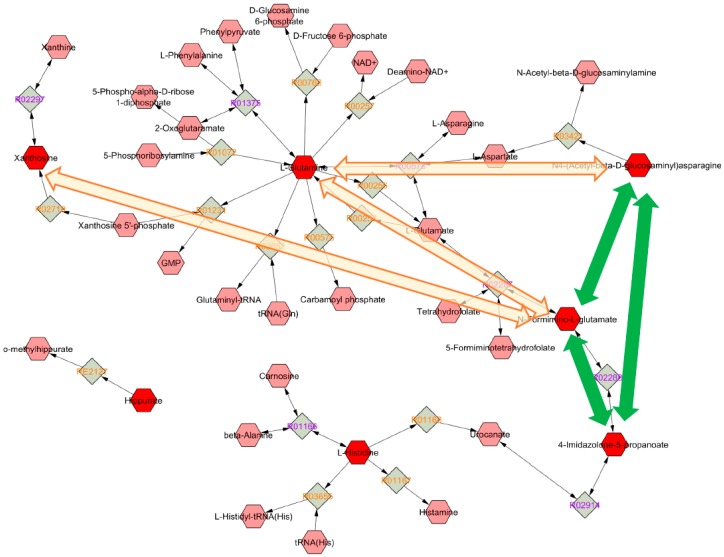
Metabolic pathway network interaction. Colored arrows represent the linear Pearson correlation of >0.3 among the identified metabolites (green: positive correlations; orange: negative correlations). The dark green lines refer to nominally significant correlations. The hexagons indicate metabolites (dark red: identified metabolites; light red: predicted metabolites), while the diamonds represent compound reactions (violet: reversible reactions; orange: irreversible reactions).

The metabolites identified were analyzed using MetScape, in order to evaluate whether there is a common pathway linking them to one another. As reported in Figure 5, glutamine exerts a pivotal role not only in glutamate metabolism, but also in histidine and alanine metabolism. The green and orange arrows shown in Figure 5 confirm this hypothesis, representing the linear Pearson correlation between the identified metabolites (green: positive correlations; orange: negative correlations).

## 4. Discussion

By using an untargeted metabolomic approach, the present pilot study conducted in healthy participants shows, besides the canonical ASA effect, putative changes in the histidine; purine; and alanine, aspartate, and glutamate metabolisms after drug treatment. The ASA inhibition of the COX enzyme in healthy participants was assessed by the measurement of TXA_2_ metabolites. The levels of serum TXB_2_ before and after ASA ingestion are comparable to those reported in the literature [22,23,24,25], and moves in parallel with the reduction of urinary levels of 11-dehydro TXB_2_.

Alongside the well-known inhibition of TXA_2_ production, we demonstrated alterations in different metabolic routes.

In the samples collected after ASA treatment, the effect on the histidine pathway is documented by the decrease of L-histidine and three major histidine catabolites: hydantoin-5-propionate, 4-imidazolone-5-propanoate, and N-formimino-L-glutamate (Figure 3). The involvement of the purine pathway was also confirmed by xanthosine reduction (Figure 3). Finally, L-glutamine and 5-aminoimidazole-4-carboxamide-1-β-D-ribofuranoside (AICAr) reduction was found, with AICAr being a link between the histidine and the purine metabolism.

Although a decrease in L-histidine and AICAr does not directly reflects changes in 5-phosphoribosyl-1-pyrophosphate (PRPP), their reduction could be related to the ASA-mediated impairment of the PPP, as ASA is known to inhibit the expression of glucose-6-phosphate dehydrogenase [26], the rate-limiting enzyme in the production of PRPP (Figure 3). Our observation of reduced urinary histidine levels after ASA treatment is in keeping with data obtained by Lewis [27] on healthy serum samples after 2 weeks of ASA ingestion using gas chromatography–mass spectrometry analysis.

Histidine is the precursor of histamine, an inflammatory mediator and an intracellular messenger involved in platelet aggregation [28]. A decrease in histamine biosynthesis after ASA treatment is thus expected [28]. The impairment of this metabolism may well play a role both in the anti-inflammatory and antiplatelet effects of ASA, reinforcing its efficacy in CVD prevention. Thus, whether the histidine reduction is associated to the concomitant decrease in the levels of histamine is unknown so far, and deserves to be elucidated in detail.

Histidine degradation leads to glutamine biosynthesis through the glutamate route. Glutamine is a key factor for cell proliferation and tumor growth [29,30]. This amino acid is metabolized within the mitochondrion through an enzymatic process termed glutaminolysis, whereby glutamine is converted to α-ketoglutarate (αKG), an intermediate of the tricarboxylic acid (TCA) cycle [31]. In highly proliferating cells, citrate produced in the TCA cycle is redirected into the cytosol for the production of NADPH and fatty acids. The production of αKG though glutaminolysis replenishes the TCA cycle [32,33,34]. In keeping with older data in this area [27,35], we report that glutamine, xantosine AICAr, 2-Oxoglutaramate, histidine, 4-imidazolone-5propanoate, N-formimino-L-glutamate, and hidantoin-5-propionate are all reduced after 7 days of ASA ingestion. L-glutamine is the nitrogen donor for the glutamine 5-phosphoribosyl-1-pyrophosphate amidotransferase, the rate-limiting enzyme in de novo synthesis of purine nucleotides [36]. Decreased levels of glutamine in the samples obtained after 7 days of ASA administration argue for an abnormal purine metabolism. Concomitant decreases in both xanthosine and AICAr (Figure 3) strengthen this hypothesis, since AICAr, the extracellular form of AICAR, is the phosphorylated precursor of purine. Cumulatively, all the changes in the metabolites that we have found appear to be related to each other (Figure 4), providing a comprehensive frame from the affected pathways and a direction to be pursued to understand chemioprotection by low-dose ASA.

Actually, in recent years, there has been increasing evidence for anticancer ASA activity. In eight studies, it the ability of ASA to delay malignancy-associated death has been demonstrated [3]. The prolongation of the period before the onset of death related to malignancy was found to be approximately five years for esophageal, pancreatic, brain, and lung cancers, and was even higher for gastric, colon, and prostate cancers [2,37]. This effect of ASA is currently explained by its ability to acetylate proteins other than COX-1 [38]. Recently, it has been demonstrated that ASA also decreases the expression of the hypoxia-inducible factor 1alpha (HIF1α), a key regulator of genes that are involved in metabolism under hypoxic conditions and a major determinant of tumor cell stabilization [39,40]. In a recent report [41], it has been shown that under normoxic conditions, HIF1α activity is significantly increased by glutamine metabolism, and is decreased by (a) acetylation via acetyl CoA synthetase or ATP citrate lyase, and (b) the presence of L-ascorbic acid, citrate, or acetyl-CoA. Interestingly, ASA significantly reduced the effect of glutamine on HIF1α [41]. The high proliferation exhibited by cancer cells requires a constant supply of nutrients [42]. To satisfy their high demand for nutrients, cancer cells undergo a metabolic reprogramming that stimulates anabolism through numerous metabolic pathways. Those pathways ultimately lead cancer cells to highly depend on specific nutrients [43].

The ability of ASA to decrease glutamine levels is of crucial importance to define the role of this drug in chemoprotection, and could pave the way to new ASA application fields.

**Figure 6:**
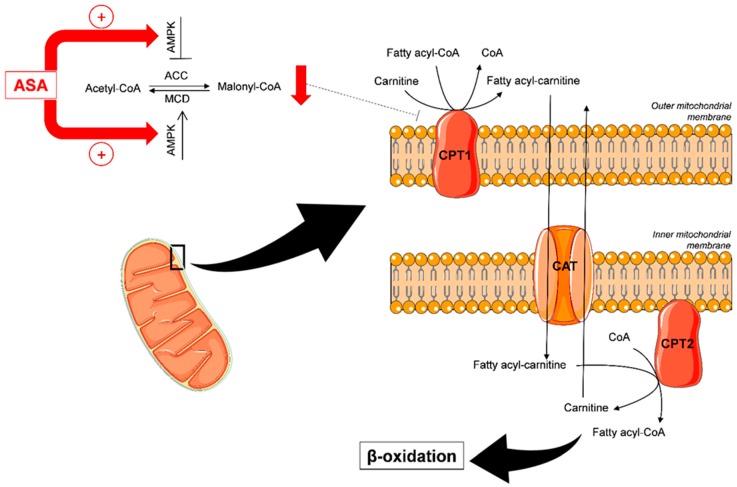
ASA induces adenosine monophosphate-activated protein kinase AMPK activity, which involves a double effect: inhibition of ACC and activation of MCD. As result, malonyl-CoA falls rapidly, because its synthesis is blocked and its degradation is enhanced. Consequently, the decrease of malonyl-CoA levels prevents the inhibition of CPT1, causing an ultimate increase in fatty acid oxidation. Thus, ASA induces the formation of fatty acyl-carnitine, catalyzing the transfer of the fatty acyl group from CoA to carnitine. Subsequently, CAT shuttles the fatty acyl-carnitine across the mitochondrial membrane. Finally, the CPT2 converts fatty acyl-carnitine back into fatty acyl-CoA, which is broken down through the *β*-oxidation catabolic process. ACC: acetyl-CoA carboxylase; AMPK: adenosine monophosphate-activated protein kinase; ASA: acetylsalicylic acid; CAT: carnitine-acylcarnitine translocase; CoA: coenzyme A; CPT1: carnitine palmitoyltransferase 1; CPT2: carnitine palmitoyltransferase 2; MCD: malonyl-CoA decarboxylase.

In our untargeted LC-QTOF-MS approach, we have also detected and putatively identified several short/medium-chain acylcarnitines: butyryl-L-carnitine, tiglylcarnitine, isovalerylcarnitine, heptanoylcarnitine, and methylglutarylcarnitine. Acylcarnitines are formed in the fatty acid (FA) metabolism to carry long-chain acyl groups of FAs into the mitochondria, where they are broken down through the *β*-oxidation pathway. In addition to inducing cancer cell death in glutamine-dependent tumors, suppression of glutaminolysis may switch cells to alternative compensatory energy sources. In an experimental model of glutamine-addicted cancers, glutaminolysis inhibition did not induce cancer cell death, and *β*-oxidation was enhanced. Accelerated lipid catabolism, together with glutaminolysis inhibition, were needed to trigger autophagy and cancer cell death [44]. As already described, ASA promotes the phosphorylation of AMP-activated protein kinase (AMPK) [6,45,46], thus leading to the reduction of malonyl–CoA, an inhibitor of the carnitine palmitoyl-transferase 1 (CPT1), with a consequent increase in FA transport (Figure 6). Thus, ASA increases the *β*-oxidation of fatty acids, as reflected by the reduced urinary excretion of the short-chain acylcarnitines in the present setting. With few exceptions [47,48], the effect of ASA on the increase of mitochondrial fatty acid oxidation has been documented in different cell lines, and interpreted as a compensatory shift from carbohydrate metabolism to FA oxidation [46]. Involvement of the *β*-oxidation pathway in the effect of a pro-drug formulation of ASA has also been reported in an animal model of hyperlipidemia [49].

We assume that an increase of methylglutarylcarnitine reflects increased peroxisomal oxidation, such a metabolite having been suggested as a marker of this process [50]. ASA is known to induce peroxisome proliferator-activated receptor alpha expression and activity [51], thus boosting *β*-oxidation. Moreover, ASA-AMPK activation switches off ATP-consuming processes, while switching on catabolic pathways that generate ATP, a major event in the fatty acid oxidation process [52].

Several studies have attempted to highlight ASA mechanisms beyond COX-1 inhibition by hypothesis-driven methods [27,35]. The broad spectrum of biochemical effects induced by a short-term ASA treatment is highlighted in the present pilot study on urine samples. This biological matrix is not affected by homeostatic regulation; it reflects physiological changes in response to metabolic dysregulation [53] and gives a time-averaged pattern, representing an attractive compartment for metabolomic studies [54]. Compared to other biological fluids, the metabolomic profile of urine—the strategy that we have chosen—provides several advantages. Urine can be noninvasively and repeatedly collected in large volumes, the protein content is absent or relatively low, and urine metabolites are thermodynamically stable [55]. On the other hand, the metabolic signature obtained through the analysis of plasma or serum represents an instantaneous readout strictly connected to the time between blood collection and ASA assumption, as a result of absorption or enzymatic inter-individual variability.

The untargeted metabolomic approach, which simultaneously measures representative metabolites derived from several pathways, may reveal unknown pathways and their potential interactions, thus avoiding the disadvantage of hypothesis-driven methods that let investigators lose the overall impact of therapy on the whole metabolism.

Our method was set up to provide an excellent range of hydrophobic separation power, and the reliability of LC-QTOF-MS analysis was ensured by the reproducibility of QC and of reference standard solution CV values. Likewise, the biological reliability of the method is documented by the occurrence of the expected ASA metabolites—i.e., salicylic acid, salicyluric acid, and 5-sulfosalicylic acid—in the samples collected after treatment. Obviously, it is important to keep in mind that this analytical approach is not exhaustive, nor does it allow the evaluation of neutral molecules or compounds that are below the limit of detection.

## 5. Conclusions

Through an untargeted metabolomics approach, we have collected data suggesting that different pathways may be affected by a short-term, low-dose ASA treatment commonly employed in clinical settings to prevent cardiovascular events. Decreased levels of urinary acylcarnitines argue for increased FA *β*-oxidation, and in turn decreased glutamine, indicating that prolonged administration of low-dose ASA may potentially exert beneficial effects beyond canonical cardiovascular protection. The data here reported also support the concept of untargeted metabolomics analysis as a major direction to be pursued in order to widely investigate treatment effects and explore new clinical applications of such a drug. Obviously, all biochemical and biological conclusions based on a pilot study with a small sample size need to be confirmed in a larger number of participants with targeted analysis. The present data provide the rationale for such studies.

## Figures and Tables

**Table 1:** List of compounds potentially modified by acetylsalicylic acid (ASA) treatment; fold changes were calculated comparing the levels before (T0) to those after treatment (T7). Compounds were selected according to the procedure described in the Statistical Methods section. VIP: variable importance in projection.

Compound	Fold Change (T0 vs. T7)	VIP Score
1,3,7-trimethyluric acid	−2.44	3.21
aspartylglycosamine	−1.79	2.90
aspartyl-isoleucine	−1.29	2.79
tiglylcarnitine	−1.30	2.53
2-methylhippuric acid	−1.69	2.18
nicotinuric acid	−1.26	2.13
2-isopropylmaleate	3.79	2.11
heptanoylcarnitine	−1.23	2.10
3-methylglutarylcarnitine	2.56	1.98
L-histidine	−1.56	1.96
xanthosine	−1.33	1.93
N-formimino-L-glutamate	−1.37	1.68
hydantoin-5-propionate	−1.35	1.66
corchoionoside B	−1.54	1.63
2-(2-phenylacetoxy)propionylglycine	1.37	1.63
prunasin	3.14	1.62
4-imidazolone-5-propanoate	−1.34	1.50
AICAr	−1.26	1.48
isovalerylcarnitine	−1.35	1.43
glycochenodeoxycholate 7-sulfate	−1.41	1.39
L-glutamine	−1.29	1.30
1-malonylamino)cyclopropanecarboxylic acid	−1.26	1.22
butyryl-L-carnitine	−1.31	1.15
piperidine	−1.65	1.08
benzeneacetamide-4-O-sulphate	−1.18	1.04

## Supplementary Materials

The following are available online at https://www.mdpi.com/2077-0383/9/1/51/s1. Figure S1: Analysis performance evaluation, Figure S2: Score plot of partial least squares-discriminant analysis (PLS-DA) models generated from the liquid chromatography-mass spectrometry (LC-MS) analysis showing the QCs (green), samples at T0 (blue) and at T7 (red) in positive ionization mode (A) and negative ionization mode (B), Table S1: List of compounds that differ between the T0 and T7 or only present at T7. Compounds were selected according to the procedure described in the statistical methods.

## Appendix A

### Reagents, Reference Materials, and Apparatus

Water, acetonitrile, formic acid, and ammonium acetate were purchased from Sigma-Aldrich (St. Louis, MO, USA), and were liquid chromatography–mass spectrometry (LC-MS) grade, as noted. Deuterated 11-dehydro-thromboxane B2 (11-DH-TXB2-d4), deuterated 8-iso-prostaglandin F2α (8-iso-PGF2α-d4), deuterated 12-hydroxyeicosatetraenoic acid (12-HETE-d8), thromboxane B2 (TXB2), and deuterated TXB2 (TXB2-d4) were purchased from Cayman Chemicals Co. (Ann Arbor, MI, USA). Reserpine and 3-nitro-tyrosine-13C9 (3-nitro-tyr-13C9) were purchased from Sigma-Aldrich; 8-hydroxy-2-deoxyguanosine-15N5 (8-OHdG-15N5) from Cambridge Isotope Laboratories, Inc. (Andover, MA, USA); and salicylic acid-d4 (SA-d4) from Santa Cruz Biotechnology (Dallas, TX, USA).

The metabolic fingerprinting, LC-MS analysis, and tandem liquid chromatography–mass spectrometry (LC-MS/MS) analysis have been performed by an ultra-high-performance liquid chromatography system (1290 Infinity series, Agilent Technologies, Santa Clara, CA, United States), coupled to a quadrupole time-of-flight (Q-TOF) mass spectrometry detector (Agilent 6550 iFunnel Q-TOF) outfitted with an electrospray ionization (ESI) source. A Zorbax Eclipse Plus C18 reverse phase column (2.1 × 150 mm, 1.8 µm, Agilent Technologies) was used.

An Accela high performance liquid chromatography System (Thermo Fisher Scientific, San Jose, CA, United States) coupled to a triple quadrupole mass spectrometer TSQ Quantum Access (Thermo Fisher Scientific), outfitted with an ESI source operating in negative mode, was used to measure TXB2 and 11-DH TXB_2_. For chromatographic separation, an XBridge C18 column (2.1 × 30 mm, 2.5 µm, Waters) was adopted.
