# Supplementary material for: Untargeted Metabolomics to Go beyond the Canonical Effect of Acetylsalicylic Acid
Source: J Clin Med. 2019 Dec 24;9(1):51. doi: 10.3390/jcm9010051 (PMC7020007; doi:10.3390/jcm9010051)
Supplement: Supplementary file 1 [file jcm-09-00051-s001.pdf]

Supplementary Information

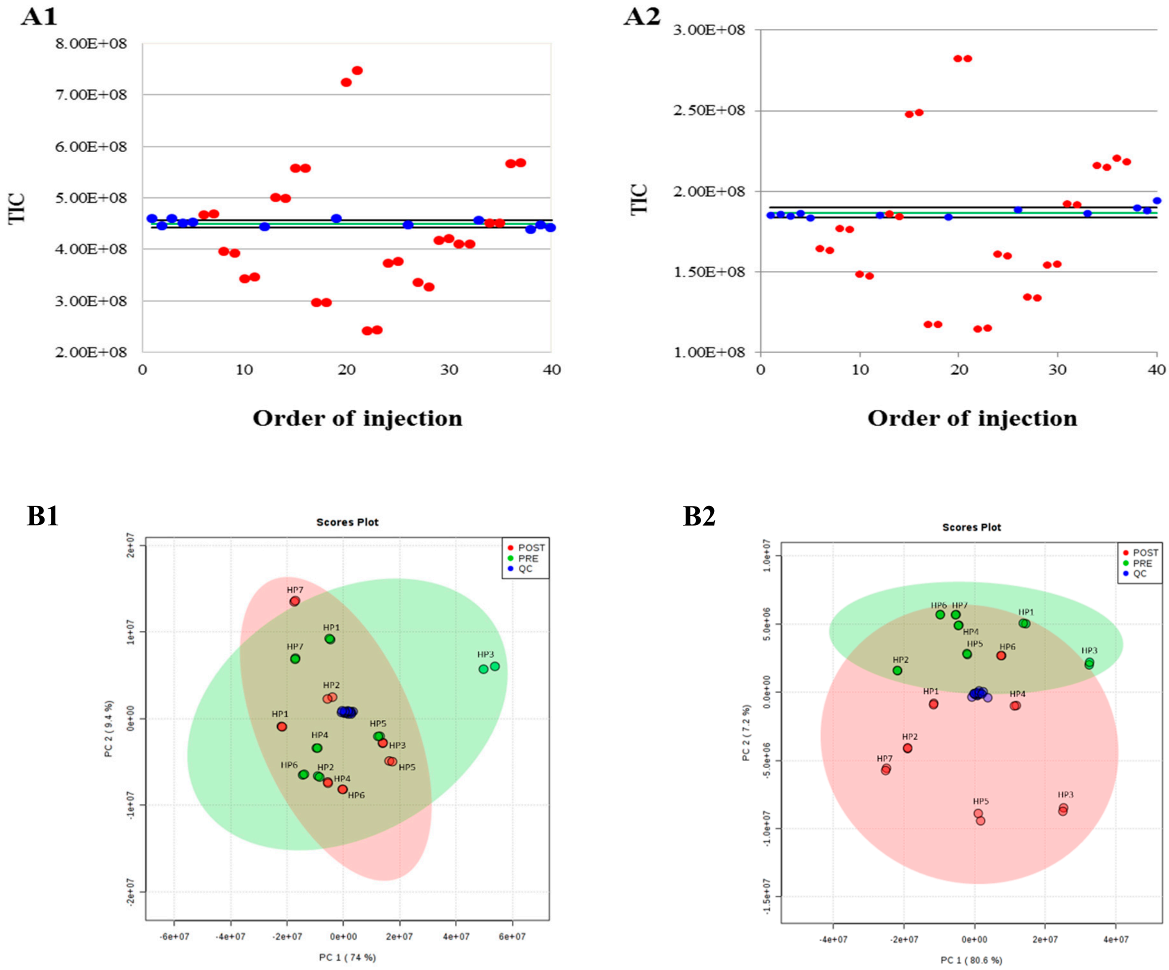

C

Reference Standard	Mass	RT	Detection mode	Mean	Std.Dev	CV%
8-OHdG-$^{15}N_5$	288.076	1.99	Positive	63664.0	5538.9	8.70
3-nitro-tyr-$^{13}C_9$	235.120	3.17	Positive	76730.5	2240.5	2.92
Reserpine	608.281	15.55	Positive	1092642.1	215462.5	19.72
SA-d_4	142.057	5.47	Negative	96795.9	3248.3	3.35
8-iso-PGF2α-d_4	358.265	15.28	Negative	67747.1	4846.5	7.15
11-DH-TXB2-d_4	372.244	15.54	Negative	93597.2	9261.8	9.89
12-HETE-d_8	328.284	17.79	Negative	75123.4	7862.0	10.46

Figure S1. Analysis performance evaluation. Trend plots showing the variation of the Total Ion Current (TIC) mean values over all observations (40 injections) in positive (A1) and negative (A2) detection mode. QCs are colored in blue and samples in red. The green line represents QCs' TIC mean value, the black lines ± standard deviation. (B) Score plot of Principal Component Analysis (PCA) models generated from the Liquid Chromatography-Quadrupole Time of Flight-Mass Spectrometry (LC-QTOF-MS) analyses showing samples at T0 (green) and at T7 (red), and quality controls (blue) in positive (B1) and negative (B2) ionization mode. (C) Assessment of reference standards added in the samples and QCs. Reference standard mass, retention time (RT), detection mode, area mean value, S.D. and coefficient of variation (CV) are expressed. 3-nitro-tyr-13C9: 3-nitro-tyrosine-13C9, 8-iso-PGF2α-d4: 8-iso-prostaglandin F2α, 8-OHdG-15N5: 8-hydroxy-2-

deoxyguanosine-15N5, 11-DH-TXB2-d4: 11-dehydro-thromboxane B2, 12-HETE-d8: 12-hydroxyeicosatetraenoic acid, SA-d4: salicylic acid- d4.

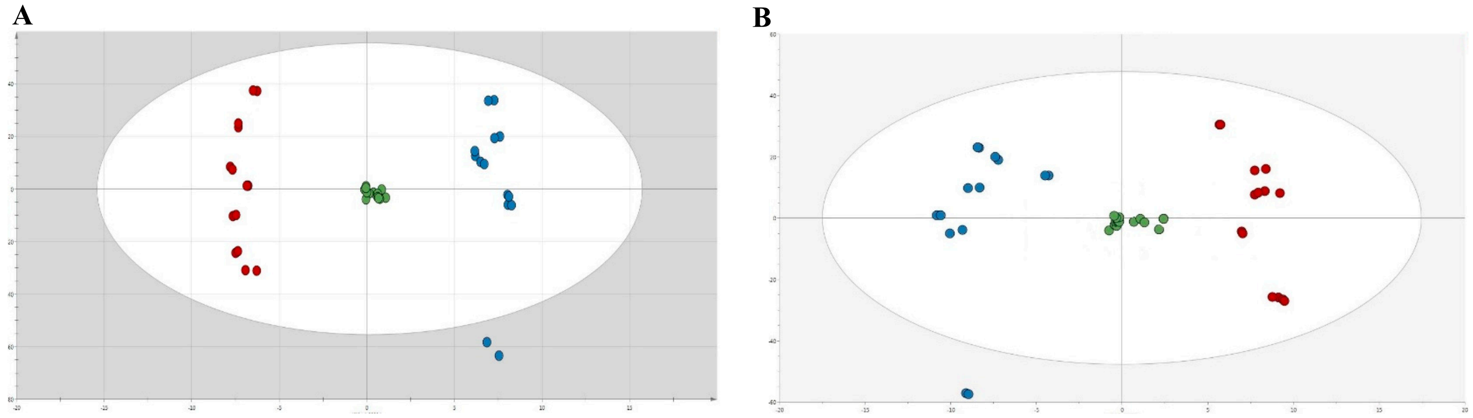

Figure S2. Score plot of partial least squares-discriminant analysis (PLS-DA) models generated from the liquid chromatography-mass spectrometry (LC-MS) analysis showing the QCs (green), samples at T0 (blue) and at T7 (red) in positive ionization mode (A) and negative ionization mode (B).

For metabolites in positive list, the explained variation parameter (R2X) was 0.51 and cross-validated predictive ability (Q2X) was 0.44. For metabolites in negative list, R2X was 0.48 and Q2X was 0.44.

In negative ionization mode, we used one predictive and two orthogonal components, and in positive ionization mode we used one predictive and three orthogonal components. The criterion for inclusion of a new component in the model was based on the resultant increase in overall R2X. For cross validation, the leave-one-out method was employed.

Supplementary Table 1. List of compounds that differ between the T0 and T7 or only present at T7. Compounds were selected according to the procedure described in the statistical methods.

Mass[a]	RT (min)[b]	Detection mode	Measured m/z[c]	Formula	ID[d]	MSI level[e]	Mass error (ppm)	Fold change (T0 *vs* T7)	VIP score	HMDB ID	Metabolism[f]
85.0891	0.98	Positive	86.0965	C5H11N	piperidine	2	1	-1.65	1.21	HMDB 34301	-
94.0421	8.88	Negative	93.0348	C6H6O *	in-source fragment		2	67.09	4.57	-	-
137.0133	13.65	Positive	138.0206	Ud	Ukn	4	-	26.10	4.91	-	-
146.0686	0.86	Negative	145.0615	C5H10N2O3	L-glutamine	2	2	-1.29	1.3	HMDB 00641	Alanine, aspartate and glutamate/Purine/Nitrogen/Aminoacyl-tRNA biosynthesis/D-glutamine and D-glutamate/Pyrimidine/Arginine and proline
151.0633	8.88	Negative	150.0561	C8H9NO2 *	in-source fragment		0	55.06	4.55	-	-
155.0690	0.91	Negative	154.0619	C6H9N3O2	L-histidine	2	2	-1.56	1.95	HMDB 00177	Histidine /Nitrogen /Aminoacyl-tRNA biosynthesis/b-Alanine/Pyrimidine
156.0529	0.90	Negative	155.0465	C6H8N2O3	4-imidazolone-5-propanoate	2	2	-1.34	1.50	HMDB 01014	Histidine
158.0578	11.38	Negative	157.0502	C7H10O4	2-isopropylmaleate	2	3	3.79	2.11	HMDB 12241	Valine, leucine and isoleucine biosynthesis
172.0475	0.88	Negative	171.0407	C6H8N2O4	hydantoin-5-propionate	2	2	-1.35	1.66	HMDB 01212	Histidine
174.0642	0.89	Negative	173.0565	C6H10N2O4	N-formimino-L-glutamate	2	1	-1.37	1.68	HMDB 00854	Histidine
180.0536	5.98	Positive	181.0608	C8H8N2O3	nicotinuric acid	2	0	-1.26	2.13	HMDB03269	Nicotinate and nicotinamide

Mass[a]	RT (min)[b]	Detection mode	Measured m/z[c]	Formula	ID[d]	MSI level[e]	Mass error (ppm)	Fold change (T0 *vs* T7)	VIP score	HMDB ID	Metabolism[f]
187.0478	1.23	Negative	186.0405	C7H9NO5	1-(malonylamino)cyclopropanecarboxylic acid	2	1	-1.26	1.22	HMDB 31700	-
190.0512	1.15	Negative	189.0896	Ud	Ukn	4	-	-1.29	1.40	-	-
191.9728	1.63	Negative	190.9655	C7H6Cl2O2 *	Ukn	4	9	2.49	2.49	-	-
193.0739	8.08	Negative	192.0666	C10H11NO3	2-methylhippuric acid	2	0	-1.69	2.18	HMDB 11723	Acyl-glycine
210.0750	8.83	Negative	209.0679	C8H10N4O3	1,3,7-trimethyluric acid	2	0	-2.44	3.21	HMDB 02123	Purine/Caffeine
213.1472	9.77	Positive	214.1546	C10H19N3O2 *	Ukn	4	2	14.44	3.73	-	-
231.0200	0.95	Negative	230.0123	C8H9NO5S	benzeneacetamide-4-O-sulphate	2	2	-1.18	1.03	HMDB 59994	-
231.1474	3.27	Positive	232.1546	C11H21NO4	butyryl-L-carnitine	2	1	-1.31	1.15	HMDB 02013	Acyl-carnitine
243.1466	6.71	Positive	244.1540	C12H21NO4	tiglylcarnitine	2	1	-1.30	2.53	HMDB 02366	Acyl-carnitine
245.1625	7.85	Positive	246.1699	C12H23NO4	isovalerylcarnitine	2	0	-1.35	1.43	HMDB 00688	Acyl-carnitine
246.1210	2.17	Positive	247.1284	C10H18N2O5	aspartyl-isoleucine	2	2	-1.29	2.79	HMDB 28756	-
248.0893	14.37	Positive	249.0969	Ud	Ukn	4	-	2.92	1.92	-	-

Mass[a]	RT (min)[b]	Detection mode	Measured m/z[c]	Formula	ID[d]	MSI level[e]	Mass error (ppm)	Fold change (T0 *vs* T7)	VIP score	HMDB ID	Metabolism[f]
248.0895	11.38	Negative	247.0822	Ud	Ukn	4	-	7.35	2.35	-	-
258.0959	2.50	Negative	257.0891	C9H14N4O5	5-aminoimidazole-4-carboxamide-1-beta-D-ribofuranoside	2	0	-1.26	1.47	HMDB 62179	Histidine/Purine
265.0966	13.66	Positive	266.1025	C13H15NO5	2-(2-phenylacetoxy)propionylglycine	2	1	1.37	1.63	HMDB 59732	Acyl-glycine
267.0741	2.90	Negative	266.0665	Ud	Ukn	4	-	51.94	4.44	-	-
273.1937	14.60	Positive	274.2008	C14H27NO4	heptanoylcarnitine	2	2	-1.23	2.10	HMDB 13238	Acyl-carnitine
274.0261	13.65	Positive	275.0337	C8H19O2PS3 *	Ukn	4	9	28.69	4.91	-	-
279.0059	13.64	Positive	280.0128	Ud	Ukn	4	-	22.86	4.91	-	-
284.0746	1.78	Positive	285.0823	C10H12N4O6	xanthosine	2	2	-1.33	1.93	HMDB 00299	Purine/Caffeine
289.1521	5.41	Positive	290.1595	C13H23NO6	3-methylglutarylcarnitine	2	1	2.56	1.98	HMDB 00552	Acyl-carnitine
289.9977	13.65	Positive	291.0058	Ud	Ukn	4	-	34.74	4.94	-	-
295.1051	14.48	Negative	294.1003	C14H17NO6	prunasin	2	7	3.14	1.61	HMDB 34934	-
304.0791	2.65	Negative	303.0718	Ud	Ukn	4	-	-1.62	2.43	-	-
315.0530	13.65	Positive	316.0608	Ud	Ukn	4	-	30.87	4.94	-	-
316.0036	13.64	Positive	317.0141	C15H9Cl2F3 *	Ukn	4	1	36.56	4.97	-	-

Mass[a]	RT (min)[b]	Detection mode	Measured m/z[c]	Formula	ID[d]	MSI level[e]	Mass error (ppm)	Fold change (T0 *vs* T7)	VIP score	HMDB ID	Metabolism[f]
318.1197	13.68	Positive	319.1268	C11H18N4O7 *	Ukn	4	7	2.66	3.60	-	-
327.0636	14.36	Positive	328.0705	Ud	Ukn	4	-	1.72	1.51	-	-
327.2408	15.63	Positive	328.2484	C18H33NO4 *	Ukn	4	1	-1.47	1.17	-	-
329.0742	4.35	Positive	330.0814	C17H13ClFN3O *	Ukn	4	3	-1.29	1.45	-	-
334.0929	13.68	Positive	335.1002	C14H14N4O6 *	Ukn	4	5	3.54	4.11	-	-
335.1302	0.90	Negative	334.1243	C12H21N3O8	aspartylglycosamine	2	4	-1.79	2.89	HMDB 00489	-
373.8337	0.77	Positive	374.8393	Ud	Ukn	4	-	-1.31	1.50	-	-
400.1723	13.83	Negative	399.1651	C19H28O9	corchoionoside B	2	2	-1.54	1.63	HMDB 30975	-
481.1398	1.12	Negative	480.1337	Ud	Ukn	4	-	-1.36	2.22	-	-
502.1428	1.25	Positive	503.1504	C24H26N2O8S*	Ukn	4	4	-1.36	1.56	-	-
529.2701	15.12	Negative	528.2627	C26H43NO8S	glycochenodeoxycholate 7-sulfate	2	2	-1.41	1.38	HMDB 02409	-
195.0534	8.88	Negative	194.0457	C9H9NO4	salicyluric acid	2	1			HMDB 00840	aspirin metabolism
138.0319	5.62	Negative	137.0243	C7H6O3	salicylic acid [@]	1	1	-		HMDB 01895	aspirin metabolism
206.0378	13.64	Positive	207.0448	Ud	Ukn [@]	4	-	-		-	-
217.0341	8.87	Negative	216.0272	Ud	Ukn [@]	4	-	-		-	-
217.9892	0.95	Negative	216.9813	C7H6O6S	5-sulfosalicylic acid	2	2	-		HMDB 11725	aspirin metabolism
412.0873	8.87	Negative	411.0801	C13H24N4O5S3*	Ukn [@]	4	9	-		-	-
428.0528	13.64	Positive	429.0598	Ud	Ukn [@]	4	-	-		-	-

428.0610	8.88	Negative	427.0533	C18H20O8S2 *	Ukn @	4	2	-	-	-
292.9932	13.64	Positive	294.0014	Ud	Ukn @	4				
